# Radiomics and machine learning for characterizing CKD-associated cortical bone texture patterns in HR-pQCT tibia scans: a slice- and patient-level methodological framework

**DOI:** 10.1093/jbmrpl/ziag123

**Published:** 2026-08-04

**Authors:** Youngjun Lee, Andy Kin On Wong, Seokkyoon Hong, Drake Dillman, Kenneth Lim, Sharon M Moe, Stuart J Warden, Ali Ghasem-Zadeh, Rachel K Surowiec

**Affiliations:** Weldon School of Biomedical Engineering, Purdue University, West Lafayette, IN 47907, United States; University Health Network, Toronto General Hospital, Toronto, ON M5G 2C4, Canada; School of Polymer Science and Engineering, Chonnam National University, Gwangju, 61186, Republic of Korea; Division of Nephrology and Hypertension, Indiana University School of Medicine, Indianapolis, IN 46202, United States; Division of Nephrology and Hypertension, Indiana University School of Medicine, Indianapolis, IN 46202, United States; Division of Nephrology and Hypertension, Indiana University School of Medicine, Indianapolis, IN 46202, United States; Department of Physical Therapy, School of Health and Human Sciences, Indiana University Indianapolis, Indianapolis, IN, United States; Departments of Endocrinology and Medicine, Austin Health, The University of Melbourne, Melbourne, VIC 3084, Australia; Weldon School of Biomedical Engineering, Purdue University, West Lafayette, IN 47907, United States

**Keywords:** HR-pQCT, CKD, bone characteristics, radiomics, machine learning classifiers

## Abstract

Chronic kidney disease (CKD) is associated with alterations in cortical bone structure and composition that contribute to increased fracture risk but are incompletely captured by standard clinical imaging, including DXA. This study evaluated whether radiomics combined with machine learning can enhance detection of CKD-related cortical bone characteristics using high-resolution peripheral quantitative computed tomography (HR-pQCT). HR-pQCT images (60.7 μm isotropic resolution; 168-slice stacks acquired at 7.3% and 30% proximal to the tibial endplate) were analyzed from 72 participants (38 non-CKD controls and 34 individuals with advanced CKD), yielding 24 192 cortical bone image slices. Cortical bone was segmented using a pretrained neural network optimized through transfer learning. Radiomic features were extracted using gray-level co-occurrence matrix (GLCM), local binary pattern (LBP), and combined GLCM+LBP feature sets, and paired with 7 machine-learning classifiers to systematically evaluate 21 feature–classifier combinations at distal and diaphyseal tibial sites. To address optimism associated with correlated slice-level data, radiomic features were aggregated per patient prior to model training and evaluated using patient-level splits (~7 test patients); this patient-level analysis constitutes the primary evaluation framework of this study. Under patient-level evaluation, classification performance was more variable across feature–classifier combinations and tibial sites, with wide CIs reflecting the limited sample size. At the slice level, hybrid GLCM+LBP features combined with XGBoost demonstrated the strongest classification performance across both tibial regions (distal AUC = 0.999; diaphyseal AUC = 0.999), providing methodological context for the systematic evaluation of all 21 feature–classifier combinations. Radiomics-derived texture features identified cortical heterogeneity that was not consistently reflected by conventional HR-pQCT metrics. These findings support radiomics as a promising methodological framework for characterizing CKD-associated cortical bone alterations while highlighting the importance of larger patient-level validation studies.

## Introduction

Individuals with CKD are much more likely to suffer from fragility fractures, with a risk up to 4 times higher compared to those without CKD, and this risk increases as the disease progresses.^1^ Unlike many other bone disorders, CKD mainly affects cortical bone, leading to higher cortical porosity, thinner cortex, and alterations in the composition of the cortical matrix itself.^2–4^ These changes reduce bone strength and increase fracture risk.^5^ In clinical practice, circulating biomarkers such as PTH and vitamin D levels are typically evaluated first as part of the fracture risk work-up before proceeding to imaging but the sensitivity and specificity for predicting underlying bone histology or fracture is poor.^6^ Although 2D DXA is commonly used to assess fracture risk, it underestimates fracture risk in patients with CKD.^4^^,^^7^^,^^8^ In CKD, BMD measurements can be misleading, as vascular calcifications, common in this population, can artificially inflate LS BMD values, and DXA cannot distinguish between cortical and trabecular compartments.^8^^,^^9^

Among skeletal sites, the tibia, particularly its distal and diaphyseal regions, represents a clinically relevant, weight-bearing bone.^10^ In this present study, these regions were analyzed to capture complementary cortical bone characteristics that differ in remodeling dynamics and mechanical loading. Lower limb fractures, including those of the tibia, are more frequently reported than upper limb fractures in patients with advanced CKD undergoing dialysis, highlighting the importance of focusing on this region for CKD-associated cortical bone assessment.^10^^,^^11^ Second-generation HR-pQCT provides detailed, compartment-specific imaging of bone microarchitecture at standard peripheral skeletal sites, including the distal and diaphyseal tibia, with a nominal isotropic voxel size of 60.7 μm and a low effective radiation dose (~5 μSv).^12–14^ Several studies have demonstrated the utility of HR-pQCT for detecting CKD-related skeletal alterations across the disease spectrum, although the specific microarchitectural endpoints identified vary by disease stage and bone compartment. For example, studies in CKD stages 2-4 have reported early trabecular deterioration prior to overt secondary hyperparathyroidism, whereas studies in dialysis-dependent CKD more consistently show reductions in cortical thickness and volumetric BMD compared with controls.^15–17^ A separate study demonstrated that HR-pQCT-derived microarchitectural measures and estimated failure load capture CKD-related skeletal deterioration even in individuals with normal or osteopenic BMD, highlighting the sensitivity of HR-pQCT to CKD-associated bone disease beyond areal BMD alone.^18^ Despite these documented abnormalities, variability in HR-pQCT outcomes across studies suggests that no single conventional metric fully captures the complexity of CKD-related bone disease. Indeed, Nickolas et al. reported that no individual cortical parameter reliably differentiated patients with fractures across anatomical sites, and Tsuji et al. observed limited separation between moderate and severe CKD stages using standard microarchitectural metrics.^15^^,^^19^ Consistent with these findings, our prior work showed that commonly reported HR-pQCT cortical outcomes, including volumetric BMD, porosity, and thickness, did not consistently distinguish dialysis-dependent CKD from non-CKD individuals.^20^^,^^21^

These observations highlight an important limitation of conventional HR-pQCT analyses: most standard metrics summarize mean density or geometry and may obscure spatially heterogeneous cortical alterations that may contribute to skeletal fragility in CKD. In a recent pilot study, we demonstrated that texture-based image features derived from HR-pQCT images could distinguish CKD-related cortical bone patterns even when conventional HR-pQCT measures overlapped between groups.^20^

Building on this foundation, the present study applies radiomics, a quantitative image analysis approach that systematically extracts spatial texture and intensity patterns from medical images,^22^ combined with machine-learning classification to characterize CKD-associated cortical bone characteristics in tibial HR-pQCT images. We hypothesize that both the radiomic feature extraction strategy and the choice of machine-learning classifier significantly influence classification performance, and that identifying an appropriate combination is necessary for robust and interpretable detection of CKD-related cortical bone texture patterns.

## Materials and methods

### Ethics

Data and images were acquired as part of the observational Function, Imaging, and Testing (FIT) Core study, which has Institutional Review Board Approval from Indiana University (Study #170755085). All participants provided written informed consent for their de-identified images and data to be used.

#### Study design

A total of 72 adults were included in this study, comprising 34 individuals with advanced CKD receiving hemodialysis and 38 age- and sex-matched non-CKD controls. The CKD participants were imaged as part of ongoing prospective studies evaluating adults aged >18 yr with advanced CKD undergoing dialysis. All CKD participants were classified as stage 5D (hemodialysis); therefore, estimated glomerular filtration rate (eGFR) was not used for classification, as it is unreliable in dialysis patients. These participants were recruited from 3 independent cohorts: 19 from the “Effects of long interdialytic intervals on cardiovascular functional capacity (ECON) trial”,^23^ 9 from the “Redefining cardiovascular risk assessment in dialysis patients (ROCK-D) study,” and 6 from the “SPARK” study (see Table S1 for inclusion and exclusion criteria). The non-CKD control subjects were recruited through the Indiana Center for Musculoskeletal Clinical Research Center FIT Core and matched by age (±5 yr) and sex.

**Figure 1:**
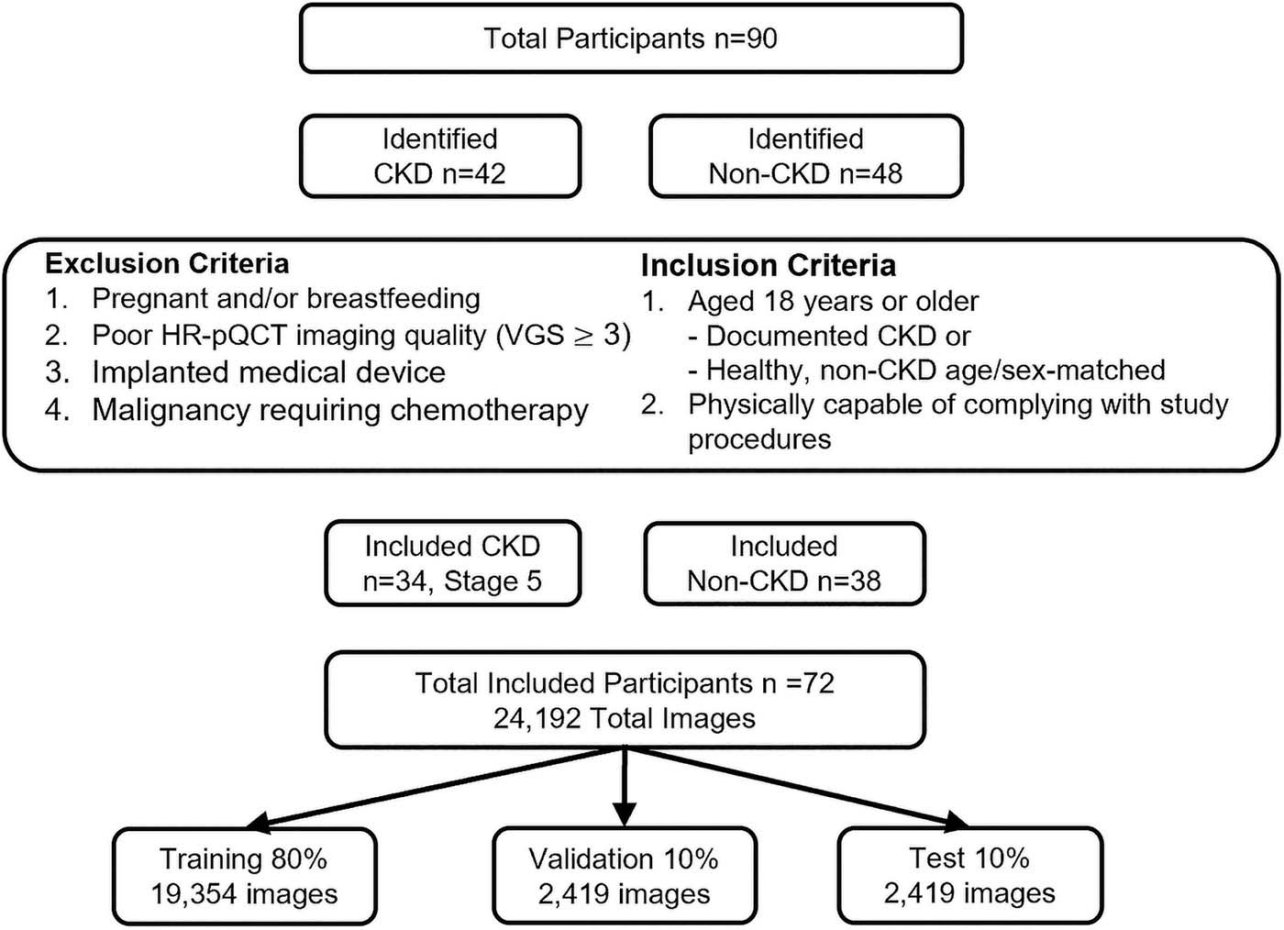
Flow diagram for image inclusion.

We utilized the existing HR-pQCT imaging with a motion artifact score (using the Visual Grading Score, VGS) of ≤3^24^ (Figure 1), inclusive of the scans from 8 CKD and 20 non-CKD participants from our prior publication.^20^ Whole-body areal BMD, T-scores, and Z-scores were obtained from DXA scans (Norland Elite; Norland at Swissray) using either a Norland Elite (Norland at Swissray) or Hologic Horizon A (Hologic Inc.) scanner. The manufacturer’s recommended imaging protocols were utilized and T- and Z-scores were calculated using the manufacturer’s sex-specific reference data. T- and Z-scores obtained using the Norland Elite scanner were converted to Hologic Horizon equivalent values using regression formulae derived by scanning 45 individuals on each scanner.

#### HR-pQCT

HR-pQCT images of the tibia were obtained from scans of the leg performed using a XtremeCT II scanner (Scanco Medical). Scan stacks were centered at 7.3% (distal) and 30% (diaphyseal) of the tibial bone length proximal to a reference line placed at the center of the distal tibial joint surface, following standardized HR-pQCT acquisition protocols to ensure anatomical consistency across participants and reproducibility between scans.^25^ The scanner operated at 68 kVp and 1.47 mA, capturing 168 slice image stacks with an isotropic voxel size of 60.7 μm. A calibration phantom provided by the manufacturer was used to ensure scanner consistency throughout the duration of the study. Bone geometry and microstructural parameters, including volumetric BMD, area, cortical and trabecular thickness, and porosity at the distal and diaphyseal tibial sites, were quantified from HR-pQCT images. Bone strength estimates, including stiffness and failure load, were subsequently derived using micro-finite element analysis (μFEA) based on the tibial scans (see Supplementary Material Section 1.1). Although radius HR-pQCT scans were acquired as part of standard site protocol, analysis was restricted to the tibia given its relevance as a weight-bearing site and to minimize heterogeneity in cortical geometry that would complicate systematic evaluation of feature–classifier pairings in this methodological framework.

### Cortical bone segmentation with deep (accelerated and transferred) learning

**Figure 2:**
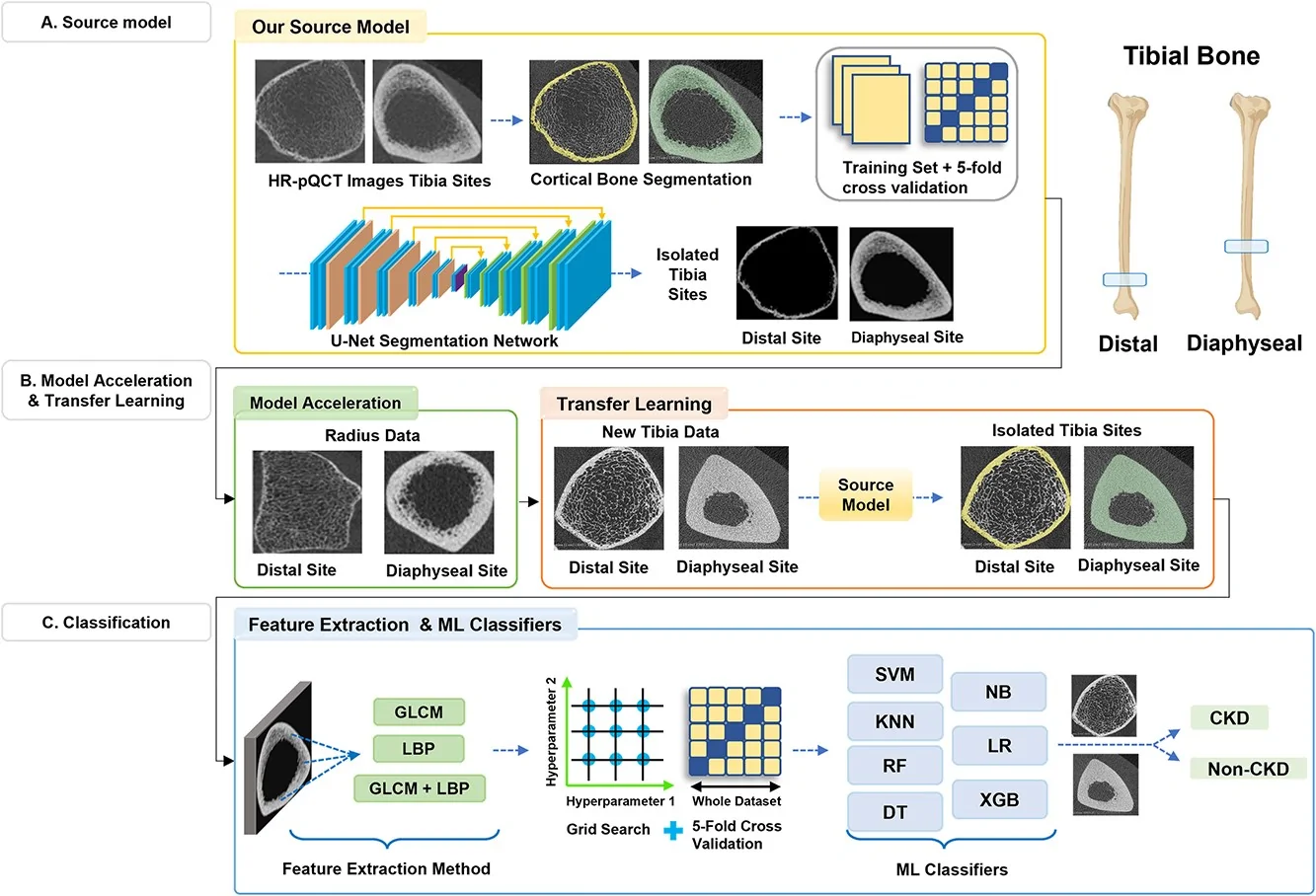
Workflow of the 2-stage tibia cortical bone analysis using HR-pQCT volumes. Workflow of the 2-stage tibia cortical bone analysis using HR-pQCT volumes. The process begins with preprocessing axial HR-pQCT tibia scans from 2 sites (distal and diaphyseal), followed by transfer learning of our validated cortices model, trained on model-accelerated radius cortical bone data. Subsequently, the predicted image was connected to radiomics process using feature extraction techniques, such as GLCM or LBP, or the combination GLCM with LBP, to extract image features in CKD and non-CKD. Then, each image feature was used to train each classifier to compare their performance and identify the best classification AI model. Abbreviations: DT, decision tree; GLCM, gray level co-occurrence matrix; KNN, K-nearest neighbors; LBP, local binary pattern; LR, logistic regression; NB, Naïve Bayes; RF, random forest; SVM, support vector machine; XGBoost, eXtreme gradient boosting.

Cortical bone was segmented away from trabecular bone compartment and surrounding soft tissue using deep learning. Two pretrained neural network-based segmentation models were previously developed using HR-pQCT images of the distal and diaphyseal tibial sites (Figure 2A).^20^ The models were subsequently retrained on a separate set of tibial images from this newly recruited cohort of participants. All HR-pQCT images were preprocessed by gray level normalization and discretization (bin widths = 0.1-0.5) using Pandas and Scikit-learn libraries in Python,^26^^,^^27^ to enhance sensitivity to subtle cortical differences associated with CKD. The final retrained segmentation model was used as the initialization for transfer learning, whereby previously learned network weights served as the starting point for further fine-tuning on tibial HR-pQCT images from the current study cohort (Figure 2B). This approach leverages learned feature representations from related HR-pQCT data to improve convergence and segmentation robustness compared with training a model from scratch.

### Textural feature extraction and outcome classification approach

#### Feature extraction methods

The initial phase involved extracting radiomic features from individual slices within the segmented cortical bone using gray-level co-occurrence matrix (GLCM) and local binary pattern (LBP) methods implemented in the scikit-learn library.^28^^,^^29^ Specifically, 24 GLCM features capturing second-order spatial relationships between gray-level intensities (contrast, homogeneity, energy, and correlation) and 26 LBP features encoding local grayscale texture patterns from uniform histogram bins were extracted. These complementary methods enable characterization of both global and fine-scale textural heterogeneity within cortical bone. The impact of these feature extraction methods on classifying CKD-related bone imaging patterns was evaluated under three models: Model 1 using only GLCM, Model 2 using only LBP, and Model 3 combining both GLCM and LBP.^28^^,^^30^^,^^31^ The extracted radiomics features and magnitudes were normalized with a mean of 0 and SD of one (*z*-score transformation).^32^

#### Machine learning classifiers

Seven different machine learning classifiers were employed: support vector machine (SVM), *k*-nearest neighbors (KNN), random forest (RF), decision tree (DT), Naïve Bayes (NB), logistic regression (LR), and eXtreme Gradient Boosting (XGBoost), each using Python’s scikit-learn package^26^ and inputting slice wise information from feature extraction methods. Among 72 participants’ distal and proximal tibia HR-pQCT images, 80% were retained for training, 10% for validation, and an additional 10% held out for testing. The data were partitioned at the patient level to avoid leakage of images from the same patient across different datasets (Figure 2C) For patient-level classification, radiomic features were aggregated per patient (mean across all slices) into a single feature vector prior to model training, and the same 80/10/10 patient-level split was applied, yielding approximately 57 training, 7 validation, and 7 test patients.

Classifier performance was evaluated based on several metrics, including accuracy, defined as (TP + TN)/(TP + TN + FP + FN), along with AUC, sensitivity, specificity, positive predictive value (PPV), and negative predictive value (NPV). Weighted averages for sensitivity and specificity were also reported to account for class imbalance. False negative rate (FNR) was calculated as FN/(FN + TP), based on the confusion matrix derived from test set predictions.

Hyperparameter tuning relevant for each classifier was performed using grid search with 5-fold cross-validation applied on the training set (see Table S2 and Supplementary Material Section 1.2) to optimize the selection of textural feature variables that result in the greatest classification performance (as guided by AUC averaged across the 5 folds). In total, 21 combinations of feature extraction and classification approaches were evaluated. The best performing 2-3 top models were validated in the 10% validation set, reporting the full set of performance metrics. The final performance of the top model was assessed on the held-out test dataset.

### Statistical analyses

Continuous data are depicted as median and interquartile (1st-3rd) range and categorical variables were presented as counts and proportions. Participant characteristics and conventional cortical HR-pQCT outcomes are reported as mean ± SD. The Shapiro–Wilk test was employed to assess normality. Continuous variables were compared using 2-sample *t*-tests or Mann–Whitney U tests, as appropriate based on data normality. Categorical variables were compared using Chi-squared tests. Statistical significance was set at *p* ≤ .05. All analyses were performed using SPSS version 28.

### Machine learning environment

The training was conducted in a computer environment with an Intel(R) Xeon(R) CPU@2.30 GHz and a GPU A100 card. All models were implemented using TensorFlow with Keras, and reproducibility was ensured through fixed randomization seeds and version tracking.

## Results

### Participant and scan characteristics

There were no significant differences between the non-CKD and CKD groups in age, height, weight, or BMI (Table 1). DXA-derived areal BMD (aBMD) and T-scores were evaluated to complement HR-pQCT measurements. The mean FN T-score was significantly lower in the CKD group compared with non-CKD participants (−1.32 ± 1.09 vs −0.72 ± 0.93, *p* = .012), indicating reduced bone density in CKD. Consistently, whole-body BMD Z-scores were also lower in the CKD group than in the non-CKD group (*p* = .03). For conventional HR-pQCT cortical measures, CKD participants exhibited 18.7% lower stiffness and 18.0% lower failure load at the distal tibia compared to non-CKD participants (both *p* = .002) (Table 2). No other distal tibia parameters differed significantly between groups. At the diaphyseal site, the only significant difference was a 9.3% reduction in cortical thickness in the CKD group (*p* = .023).

**Table 1:** Participant characteristics.

Characteristics	Mean (SD), *n* (%)		
	Non-CKD (*n* = 38)	CKD (*n* = 34)	*p*-value
**Age (yr)**	54 *±* 14.70	52.34 *±* 13.35	.58
**Sex (female)**	18 (47%)	14 (41%)	.79
**Height (cm)**	171.22 *±* 10.18	169 *±* 11.00	.61
**Weight (kg)**	79.06 *±* 14.70	84.06 *±* 17.55	.20
**BMI (kg/** ${\mathbf{m}}^{\mathbf{2}}$**)**	26.84 *±* 3.76	29.26 *±* 6.58	.07
**Whole body aBMD**	0.980 *±* 0.128	0.976 *±* 0.129	.197
**Whole body *z*-score**	0.39 *±* 1.14	−0.30 *±* 1.17	.009
**Whole body *t*-score**	0.03 *±* 1.24	0.74 *±* 1.48	.021
**Femoral neck aBMD**	0.834 *±* 0.124	0.793 *±* 0.153	.211
**Femoral neck *z*-score**	0.19 *±* 0.85	−0.49 *±* 0.97	.002
**Femoral neck *t*-score**	−0.72 *±* 0.93	−1.32 *±* 1.09	.012

The *p*-values between non-CKD and CKD patients were determined utilizing *t*-tests or Chi-squared tests. Values are shown as means ± SD or as counts (percentages).

**Table 2:** Conventional HR-pQCT cortical outcomes.

Bone	Location	Cortical outcome	Mean ± SD			
			CKD	Non-CKD	*p*-value	Cohen’s *d*
**Tibia**	Distal	Ct.vBMD (mgHA/cm^3^)	862.24 *±* 94.18	892.34 *±* 73.65	.163	0.360
		Ct.Ar (mm)	132.36 *±* 30.36	145.51 *±* 35.36	.126	0.396
		Ct.Th (mm)	1.47 *±* 0.31	1.59 *±* 0.35	.158	0.365
		Ct.Pm (mm)	108.85 *±* 11.58	108.91 *±* 12.48	.985	0.005
		Ct.Po (%)	3.09 *±* 1.55	3.09 *±* 1.60	.997	0.001
		Ct.PoDm (mm)	0.22 *±* 0.03	0.24 *±* 0.03	.064	0.481
		Stiffness (kN/mm)	176.40 *±* 45.85	216.99 *±* 59.50	.002	0.751
		Failure load (kN)	15.26 *±* 3.84	18.61 *±* 4.89	.002	0.747
	Diaphyseal	Ct.vBMD (mgHA/cm^3^)	1011.86 *±* 45.75	1027.43 *±* 23.80	.095	0.440
		Ct.Ar (mm)	283.06 *±* 89.49	295.02 *±* 50.06	.408	0.217
		Ct.Th (mm)	5.44 *±* 1.03	6.00 *±* 0.81	.023	0.608
		Ct.Pm (mm)	82.28 *±* 8.46	80.68 *±* 7.21	.435	0.204
		Ct.Po (%)	1.23 *±* 0.98	1.02 *±* 0.52	.298	0.273
		Ct.PoDm (mm)	0.21 *±* 0.04	0.22 *±* 0.03	.089	0.449
		Stiffness (kN/mm)	301.60 *±* 65.11	314.51 *±* 54.48	.418	0.214
		Failure load (kN)	26.17 *±* 5.77	27.23 *±* 4.72	.351	0.242

Comparison of the conventional cortical HR-pQCT between the non-CKD and CKD groups’ results. *p* < .05 means statistical significance. Micro-FEA outcomes of stiffness and failure load considered both trabecular and cortical bone compartments. Failure load values are reported as absolute magnitudes for clarity, since the directionality of the simulated compressive load is not clinically meaningful. Abbreviations: Ct.Ar, cortical area; Ct.Pm, cortical perimeter; Ct.Po, cortical porosity; Ct.PoDm, cortical pore diameter; Ct.Th, cortical thickness; Ct.vBMD, cortical volumetric bone mineral density.

### Evaluation of the classification models

The optimal hyperparameters were derived by hyperparameter tuning in the training set: KNN with *n*_neighbors = 5, DT with max_depth = 10, RF with *n*_estimators = 200 and max_depth = 10, and XGBoost with *n*_estimators = 300, max_depth = 5, and learning_rate = 0.1. The hyperparameter settings for the remaining classifiers are provided in Supplementary Material Section 1.3. All results reported in this section reflect classifier performance on the held-out test set, after model selection using the validation set.

#### The GLCM feature extractor with machine-learning classifiers

Using the held-out test set, the top 3 classifiers using feature extractor GLCM on the distal tibia region are XGBoost, RF, and DT, which were the same as in the case for the diaphyseal tibia region (Tables S3 and S5, Figures S5 and S7). Among the 3 classifiers using GLCM features, XGBoost achieved the highest AUC-ROC at both tibial sites. In the distal tibia, XGBoost showed an accuracy of 0.900 and AUC-ROC of 0.974, which were 0.004 and 0.003 higher than RF (accuracy: 0.896, AUC-ROC: 0.971), and 0.037 and 0.106 higher than DT (accuracy: 0.863, AUC-ROC: 0.868). At the diaphyseal tibia, XGBoost reached an accuracy of 0.979 and AUC-ROC of 0.997, exceeding RF by 0.006 and 0.001 (RF: 0.973, 0.996), and DT by 0.031 and 0.049 (DT: 0.948, 0.948). The FNR of incorrectly classifying CKD images as non-CKD for distal and diaphyseal tibia was (1) XGBoost: 12.49% and 2.76%, (2) RF: 13.18% and 3.27%, and (3) DT: 14.81% and 5.08%, respectively (Figures S1 and S3).

#### The LBP feature extractor with machine-learning classifiers

The top 3 classifiers using feature extractor LBP on the distal tibia region are XGBoost, RF, and KNN, which is the same in the case of the diaphyseal tibia region (Tables S4 and S6, Figures S6 and S8). Among the 3 classifiers using LBP features, XGBoost achieved the highest performance across both tibial sites. In the distal tibia, XGBoost reached an accuracy of 0.989 and AUC-ROC of 0.999, which were 0.003 higher in accuracy than RF (0.986) and 0.010 higher than KNN (0.979), while the AUC-ROC was the same as both RF and KNN (0.999). At the diaphyseal tibia, XGBoost achieved an accuracy of 0.994 and AUC-ROC of 0.999, which was 0.001 higher in accuracy than RF (0.993) and 0.004 higher than KNN (0.990), while the AUC-ROC was the same as RF and 0.001 higher than KNN (0.998). The FNR of incorrectly classifying CKD images as non-CKD for distal and diaphyseal tibia is (1) XGBoost: 1.21% and 0.52%, (2) RF: 1.29% and 0.95%, and (3) KNN: 1.98% and 0.52%, respectively (Figures S2 and S4).

#### The GLCM and LBP hybrid feature extractor with machine-learning classifiers

**Figure 3:**
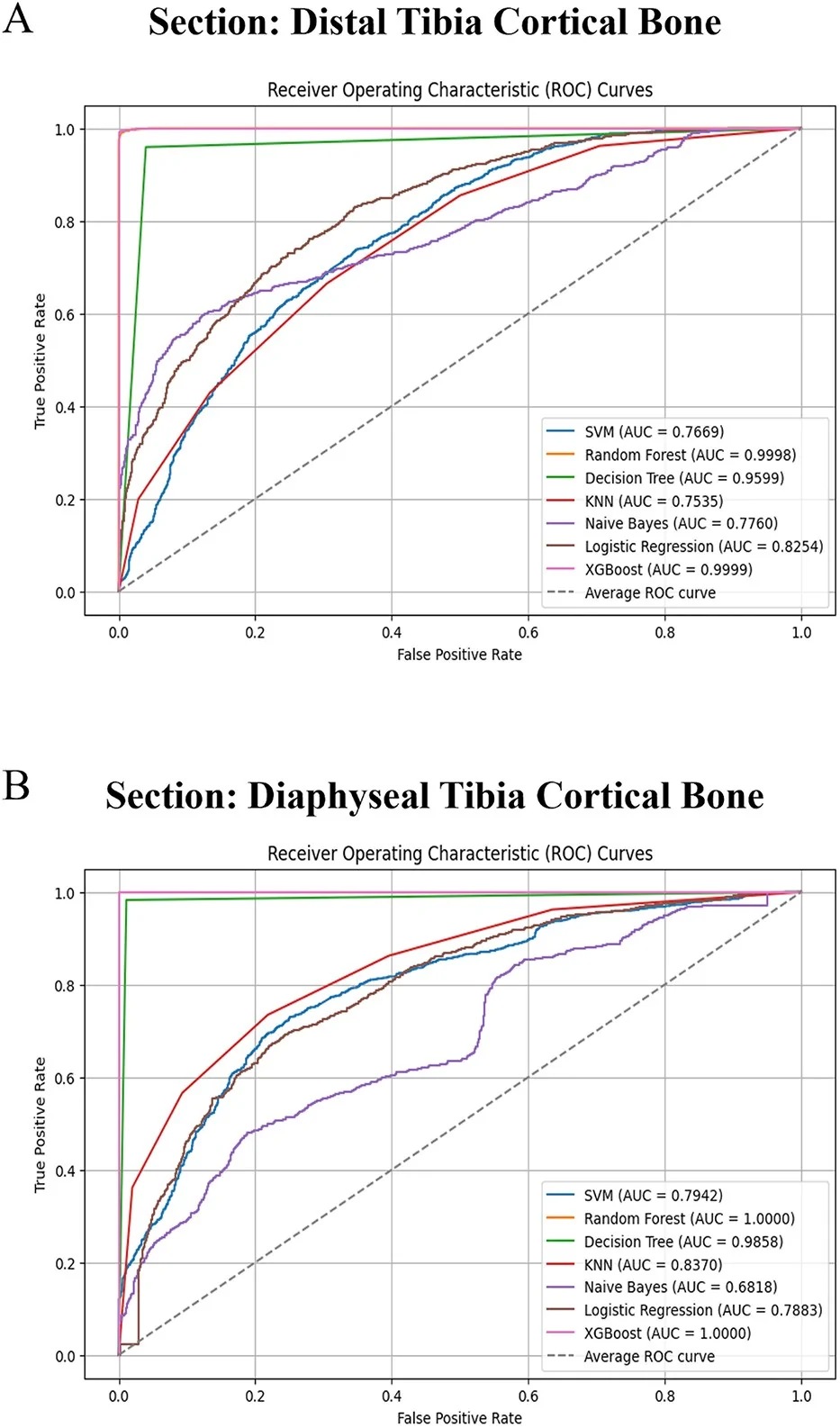
AUC-ROC curves for discriminating CKD patients from non-CKD participants using HR-pQCT cortical bone radiomics. (A) Distal tibia and (B) diaphyseal tibia cortical regions were analyzed across various classification models ranging from SVM to XGBoost, using combined GLCM + LBP radiomic features derived from transfer deep learning with model acceleration. Abbreviations: AUC-ROC, area under the curve of the receiver operating characteristic; DT, decision tree; KNN, K-nearest neighbors; LR, logistic regression; NB, Naïve Bayes; RF, random forest; SVM, support vector machine; XGBoost, eXtreme gradient boosting.

The best-performing machine learning classifiers among the 21 tested combinations were the XGBoost classifiers fused with hybrid GLCM and LBP feature extraction, achieving AUC-ROC of 99.20% and 99.99% for the distal tibia, and 99.99% and 99.80% for the diaphyseal tibia in the held-out test set (Tables 3 and 4). The corresponding AUC-ROC values were 0.999 for both RF and XGBoost at the distal and diaphyseal tibia sites, compared to 0.959 and 0.986 for DT, respectively (Figure 3A and B).

**Table 3:** Machine learning classifier performance on GLCM and LBP-processed tibia distal cortical compartment on the held-out test set.

GLCM + LBP	GLCM + LBP and tibia distal cortical bone classification performance metrics						
	Sensitivity	Specificity	FNR	PPV	NPV	Accuracy	AUC-ROC
**SVM**	0.748	0.630	0.252	0.651	0.730	0.686	0.766
**RF**	0.991	0.993	0.008	0.992	0.992	0.992	0.999
**DT**	0.960	0.960	0.040	0.957	0.963	0.960	0.959
**KNN**	0.665	0.695	0.335	0.668	0.692	0.681	0.754
**NB**	0.794	0.485	0.206	0.587	0.718	0.633	0.776
**LR**	0.675	0.794	0.325	0.752	0.726	0.737	0.825
**XGBoost**	0.992	0.999	0.007	0.999	0.993	0.996	0.999

The FNRs were directly computed from the confusion matrices using the formula FN/(FN + TP), not by subtracting the rounded sensitivity values, such as RF, DT, and XGBoost, ensuring greater numerical accuracy than using 1 − sensitivity derived from the table. Abbreviations: AUC-ROC, area under the curve of the receiver operating characteristic; DT, decision tree; FNR, false negative rate; KNN, K-nearest neighbors; LR, logistic regression; NB, Naïve Bayes; NPV, negative predictive value; PPV, positive predictive value; RF, random forest; SVM, support vector machine; XGBoost, eXtreme gradient boosting.

**Table 4:** Machine learning classifier performance on GLCM and LBP-processed tibia diaphyseal cortical compartment on the held-out test set.

GLCM + LBP	GLCM + LBP and tibia diaphyseal cortical bone classification performance metrics						
	Sensitivity	Specificity	FNR	PPV	NPV	Accuracy	AUC-ROC
**SVM**	0.790	0.661	0.210	0.682	0.773	0.723	0.794
**RF**	0.999	0.999	0.001	0.999	0.999	0.999	0.999
**DT**	0.983	0.989	0.017	0.988	0.984	0.986	0.986
**KNN**	0.735	0.782	0.256	0.756	0.762	0.759	0.837
**NB**	0.816	0.448	0.184	0.578	0.724	0.625	0.682
**LR**	0.676	0.768	0.324	0.729	0.720	0.724	0.788
**XGBoost**	0.999	0.998	0.001	0.998	0.999	0.999	0.999

The FNRs were directly computed from the confusion matrices using the formula FN/(FN + TP), not by subtracting the rounded sensitivity values, such as RF, DT, and XGBoost, ensuring greater numerical accuracy than using 1 − sensitivity derived from the table. Abbreviations: AUC-ROC, area under the curve of the receiver operating characteristic; DT, decision tree; FNR, false negative rate; KNN, K-nearest neighbors; LR, logistic regression; NB, Naïve Bayes; NPV, negative predictive value; PPV, positive predictive value; RF, random forest; SVM, support vector machine; XGBoost, eXtreme gradient boosting.

**Figure 4:**
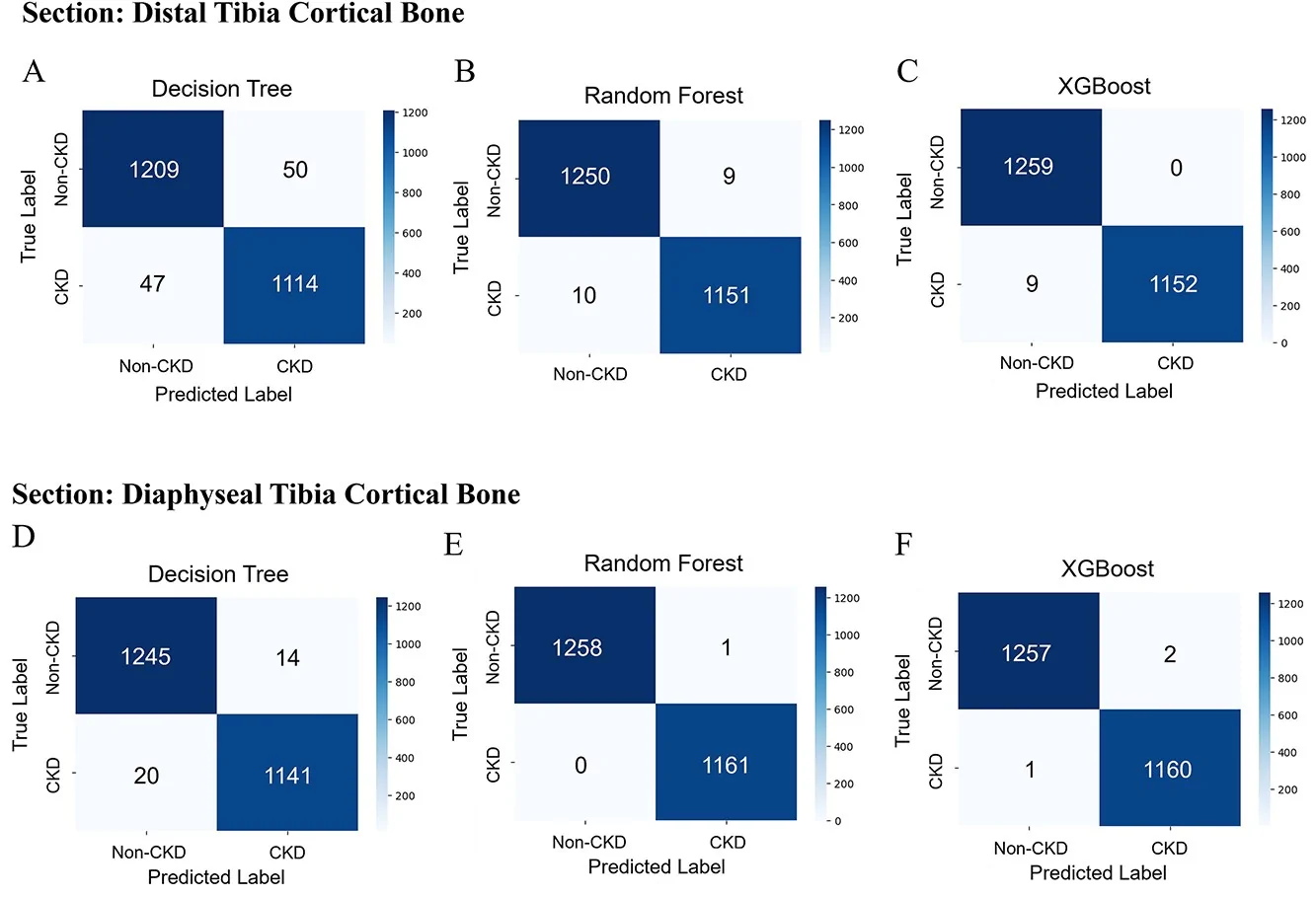
Confusion matrices for binary (non-CKD and CKD) classification using the top three ML classifiers from (A) decision tree, (B) random forest, and (C) XGBoost based on distal tibia cortical bone, and (D) decision tree, (E) random forest, and (F) XGBoost based on diaphyseal tibia cortical bone. All models were trained and evaluated using combined GLCM + LBP features on the hold-out test set. The numbers within each box represent the number of images classified into each category. The hold-out test set evaluates how well the trained model performs on entirely new, unseen data, providing an estimate of its real-world predictive ability.

In the distal tibia sites using classifier XGBoost, the hybrid approach combining GLCM and LBP features achieved the highest classification accuracy (0.996), outperforming models using GLCM alone (0.900) or LBP alone (0.989). Also, the XGBoost using the hybrid method outperformed other ML classifiers across all evaluation metrics shows superior performance over all other combinations of GLCM- or LBP-feature extractor, reducing misclassification of CKD images from an FNR of 12.49% (GLCM) and 1.21% (LBP) to 0.78% (Hybrid) for the distal tibia, as depicted in Figures 4A-C and S9.

At the diaphyseal sites using classifier XGBoost, the hybrid approach of the combined feature extractors GLCM and LBP overall outperformed other combinations using the feature extractor GLCM or LBP, especially almost similar to the results derived from the classifier RF with the combined feature extractor GLCM and LBP in terms of evaluation metrics, reducing misclassification of CKD images from FNR: of 2.76% (GLCM) and 0.52% (LBP) to 0.09% (hybrid) for the diaphyseal tibia, as shown in Figures 4D-F and S10. Using the optimal XGBoost model with fused GLCM and LBP features, a small number of images were misclassified between CKD and non-CKD groups. Specifically, 9 CKD images were misclassified as non-CKD at the distal tibia site, whereas at the diaphyseal tibia site, 2 non-CKD images were misclassified as CKD and 1 CKD image was misclassified as non-CKD (Figures 4C and F, S9, and  S10).

#### Patient-level classification performance

At the patient level, the best-performing classifiers varied by feature extractor and tibial site (Table S7, Figure S11). At the distal tibia, RF achieved the highest AUC using GLCM features (AUC = 1.000, sensitivity = 1.000, and specificity = 0.750), RF also led with LBP features (AUC = 1.000, sensitivity = 1.000, and specificity = 0.500), and KNN achieved the highest performance with combined GLCM+LBP features (AUC = 1.000, sensitivity = 1.000, and specificity = 1.000). With 7 test patients, an AUC of 1.000 at the distal tibia is a consequence of the small test set size rather than a reflection of strong discriminative performance. At the diaphyseal tibia, NB achieved the highest AUC with GLCM features (AUC = 0.812, sensitivity = 0.750, and specificity = 0.500), DT led with LBP features (AUC = 0.875, sensitivity = 1.000, and specificity = 0.750), and NB performed best with combined GLCM+LBP features (AUC = 0.812, sensitivity = 1.000, and specificity = 0.250). Confidence intervals were wide across all combinations, reflecting the small test set size (*n* ≈ 7-8 patients). The limited and variable patient-level performance across both tibial sites likely reflects the combined effect of small sample size and regional differences in cortical texture characteristics.

## Discussion

This study extends our previous proof-of-concept analysis in which radiomics features derived from HR-pQCT images can differentiate cortical bone in individuals with and without CKD.^20^ To build on these findings, we increased the sample size to 34 individuals with CKD receiving hemodialysis and 38 age- and sex-matched non-CKD controls and evaluated 21 cross-combinations of 3 feature extractors and 7 machine-learning classifiers to assess how feature–classifier pairing influences CKD-related cortical texture discrimination at 2 tibial sites. Gray-level co-occurrence matrix and LBP capture different aspects of image texture, and the seven classifiers vary in how they handle feature interactions and nonlinearity, so evaluating combinations rather than a single pipeline was necessary to understand which pairings work and under what conditions.

XGBoost and other tree-based classifiers performed consistently well, likely because they handle nonlinear relationships and high-dimensional feature spaces without requiring extensive preprocessing.^33^ XGBoost in particular has shown utility across medical imaging applications, including oncology and cardiovascular risk prediction.^34^^,^^35^ In the present study, LBP-based features produced stronger classification consistency than GLCM features alone in terms of PPV, NPV, and ROC, consistent with prior reports local texture description.^36^ Tree-based models—especially RF, DT, and XGBoost—showed superior performance overall, though KNN showed strong results specifically when paired with LBP features, suggesting that some feature–classifier combinations interact in ways that go beyond general classifier rankings.

Combining GLCM and LBP improved discrimination when paired with ensemble classifiers. The two descriptors capture different things: GLCM reflects second-order spatial statistics, such as contrast and correlation, which may track broader density organization, while LBP encodes local grayscale variation tied to fine-scale texture. In advanced CKD, cortical bone deterioration involves both diffuse and focal alterations, including thinning, porosity, and matrix-level heterogeneity, suggesting that no single texture descriptor is sufficient.^37^ Using both together provides a more complete picture of CKD-associated cortical bone patterns. Gray-level co-occurrence matrix and LBP are also interpretable by design, which matters for early-stage biomarker work where understanding what drives classification is as important as performance.^22^^,^^38^ Slice-level evaluation enabled comparison across all 21 combinations; patient-level analysis then tested whether that discrimination holds beyond within-scan texture coherence.^39^

While the hybrid GLCM–LBP pairing with ensemble classifiers demonstrated superior performance in this cohort, the optimal combination of feature descriptor and classifier is likely context-dependent. Performance may vary according to disease stage, imaging protocol, and cohort composition; therefore, no single universally optimal feature–classifier pairing can be advocated. Prior studies in diverse imaging domains, including brain tumor, thyroid nodule, and general texture classification, have shown similar benefits from hybrid radiomics approaches, highlighting their ability to capture both global texture dependencies and local micro-patterns.^31^^,^^41^^,^^42^

Prior work applying radiomics to HR-pQCT has demonstrated the potential of texture-based analysis for skeletal health. For example, Lu et al.^30^ applied a RF model using 3D LBP features from tibial HR-pQCT images to predict fractures in community-dwelling older adults. Their model incorporated DXA-derived FN BMD and clinical variables, such as age, sex, BMI, and dietary calcium, and achieved a sensitivity of 0.83 and a specificity of 0.74. However, their analysis was limited to a single feature extractor (LBP), one classifier (RF), and a general fracture risk outcome. In our earlier pilot study involving a smaller CKD cohort, texture-based radiomics features extracted via GLCM and classified using XGBoost differentiated CKD-associated cortical bone patterns using image-only inputs achieving strong sensitivity and specificity.^20^ While encouraging, that study was limited by sample size and methodological scope. The present work builds on both by comparing multiple feature–classifier combinations, showing that paired GLCM+LBP with ensemble classifiers produced more consistent discrimination across tibial sites than either descriptor alone, and that this holds even when analysis is pushed to the patient level.

Several limitations should be acknowledged. Slice-level classification was supplemented with patient-level analysis in which radiomic features were aggregated per patient prior to model training, avoiding the optimism inherent in probability averaging of correlated within-scan data. At the patient level, performance was more variable, particularly at the diaphyseal tibia, where AUC ranged from 0.188 to 0.875 across feature–classifier combinations. Confidence intervals were wide throughout, reflecting the honest inferential capacity of approximately 7 test patients rather than a methodological failure. Several combinations showed high sensitivity but near-zero specificity, indicating bias toward the CKD class that likely reflects limited training sample size rather than genuine discrimination. The classifier rankings from slice-level analysis did not replicate at the patient level: XGBoost with hybrid GLCM+LBP led at both tibial sites in slice-level evaluation, but KNN with GLCM+LBP performed best at the distal tibia and DT with LBP at the diaphyseal tibia when evaluated at the patient level. This inconsistency has a direct practical implication: slice-level benchmarking cannot be assumed to reliably guide classifier selection for patient-level clinical use. The reversal in site-level rankings between analysis levels, the diaphyseal tibia outperformed the distal tibia at the slice level but not at the patient level, may reflect that within-patient texture coherence at the diaphyseal site, which drives high slice-level AUC, does not translate to stronger between-patient discrimination. Taken together, these findings indicate that both levels of analysis are needed to characterize classifier behavior across tibial sites, and that larger cohorts are required before any feature–classifier pairing can be recommended with confidence.^43–45^

This analysis was restricted to cortical bone at the tibia. This was a deliberate starting point: advanced CKD is predominantly characterized by cortical deterioration, and restricting the initial evaluation to the cortical compartment allowed for a controlled methodological comparison. Whether the observed classification performance reflects cortical-specific alterations or broader skeletal texture differences involving the trabecular compartment is an important open question that this study cannot address. The same segmentation pipeline produces trabecular masks as a standard output, and compartment-comparative analysis is a clear priority; however, doing so at the patient level requires sample sizes the present cohort cannot support. The study did not include fracture outcomes, bone biopsies, or other validated fragility endpoints. The findings reflect CKD-associated cortical imaging characteristics rather than fracture risk prediction. In the absence of validation against histological measures, biochemical turnover markers, or established HR-pQCT parameters, these radiomic signatures should be considered hypothesis-generating imaging markers rather than defined structural or pathophysiological patterns. Sample size was small and drawn from a single center, which raises overfitting risk. Five-fold cross-validation during model training helped stabilize performance estimates.^40^

The control group consisted of age- and sex-matched non-CKD volunteers, which may increase separability between groups compared to more clinically similar skeletal populations. Future studies should include comparator groups matched not only on age and sex, but also on DXA-defined skeletal status or conventional HR-pQCT cortical measures, such as non-CKD individuals with osteopenia, osteoporosis, or fracture history The cohort was limited to advanced CKD, and it remains unclear whether radiomics can detect subtler cortical changes at earlier disease stages (stages 2-4), where conventional HR-pQCT metrics have shown less consistent discrimination.^20^ Cortical bone segmentation was performed using a 2D pretrained neural network,^39^ and while 2D approaches may perform well in certain tasks, they may limit full characterization of 3D structural complexity. Although deep learning offers powerful alternatives, we prioritized interpretable machine learning-based features to support transparent evaluation of imaging characteristics.^38^

This study evaluated 21 combinations of radiomic feature extractors and machine-learning classifiers for detecting cortical bone differences in adults with advanced CKD versus age- and sex-matched controls using tibial HR-pQCT images. Which feature–classifier combination is used matters: hybrid GLCM+LBP features paired with ensemble classifiers showed the most consistent discrimination across both tibial sites at the slice level, while patient-level analysis revealed that these rankings do not hold uniformly, with wide CIs reflecting the limits of a small test cohort. Radiomics captured cortical texture differences that conventional HR-pQCT metrics did not consistently detect. Larger studies with patient-level designs are needed to confirm which combinations generalize, but the approach is viable and the groundwork is laid for applying texture-based imaging to CKD-related skeletal assessment.

## Supplementary Material

Supplementary_v5-YJL_ziag123

## Data Availability

The data that support the findings of this study are available from the corresponding author upon reasonable request.
