# Supplementary material for: Radiomics and machine learning for characterizing CKD-associated cortical bone texture patterns in HR-pQCT tibia scans: a slice- and patient-level methodological framework
Source: JBMR Plus. 2026 Aug 4;10(10):ziag123. doi: 10.1093/jbmrpl/ziag123 (PMC13552499; doi:10.1093/jbmrpl/ziag123)
Supplement: Supplementary file 1 [file supplementary_v5-yjl_ziag123.docx]

**Radiomics and Machine Learning for Characterizing CKD-Associated Cortical Bone Texture Patterns in HR-pQCT Tibia Scans: A Slice- and Patient-Level Methodological Framework**

Youngjun Lee *et al.*

*Corresponding author. Email: [rsurowie@purdue.edu](mailto:rsurowie@purdue.edu)

This PDF File includes:

Details in High-resolution peripheral quantitative computed tomography (HR-pQCT)

Tables. S1 to S7

Figures. S1 to S11

Supplemental References

**1.1. High-resolution peripheral quantitative computed tomography (HR-pQCT)**

HR-pQCT (XtremeCT II, Scanco Medical, Bruttisellen, Switzerland) was acquired on the participants’ non-dominant leg, as previously described (1). Bone length was measured in triplicate using a segmometer (Realmet Flexible Segmometer, NutriActiva, Minneapolis, MN) as described elsewhere (2). Participants were scanned while in supine on a movable treatment plinth with the limb of interest immobilized using a manufacturer-provided carbon fiber cast. The scanner operated at 68 kVp and 1.47 mA, capturing 168 slices (equivalent to 10.2 mm of bone length) with a voxel size of 60.7 μm. Scanner stability was verified throughout the duration of data collection by scanning phantoms with inserts of known density and volume, per manufacturer instructions. Scan stacks were positioned at 7.3% and 30% of the bone length proximal to the tibia reference line. Scans were reconstructed using manufacturer-supplied algorithms. Once the cortical bone was extracted, the following cortical (Ct) outcomes were recorded at the diaphyseal and distal tibia site: volumetric BMD (Ct.vBMD, mgHA/cm^3^), area (Ct.Ar, mm^2^), thickness (Ct.Th, mm), and porosity (Ct.Po, %). µFE analysis (Scanco Medical FE software version 1.16) was used to estimate stiffness (kN/mm) and failure load (kN) using a pixel-wise assigned modulus of 10 GPa and a Poisson’s ratio of 0.3 and evaluated under axial compression, as previously described (3).

**1.1.1 Participant characteristics**

Participant height (measured to the nearest 0.1 cm) and weight (measured to the nearest 0.1 kg) were obtained without shoes, using a calibrated stadiometer (Seca 264; Seca GmbH & Co., Hamburg, Germany) and scale (MS140–300; Brecknell, Fairmont, MN), respectively. Whole-body areal bone mineral density (aBMD) were evaluated through whole-body DXA (Norland Elite; Norland at Swissray, Fort Atkinson, WI).

**Supplemental Table 2** shows the criteria for chronic kidney disease based on eGFR. Chronic Kidney Disease (CKD) is a progressive condition characterized by the gradual loss of kidney function over time. It is typically categorized into stages based on the estimated glomerular filtration rate (eGFR), which is a measure of how well the kidneys are filtering waste products from the blood (4). Based on this criterion, CKD dataset is exported from HR-pQCT and used in this study.

**1.2. Grid Search Hyperparameter Optimization and Model Evaluation Procedure**

As described in Section 2.3.2, we applied grid search with 5-fold cross-validation to optimize model performance using GridSearchCV from scikit-learn (v0.21, Python 3.7) (5). Each classifier was paired with a predefined set of hyperparameters based on standard practice and empirical testing. During tuning, the training and validation set (80% and 10% of the data, split at the patient level) was divided into five folds. Models were trained and validated across these folds, and the configuration with the highest average accuracy was selected as the final model. The optimized model (best_estimator_) was then retrained on the full training data and evaluated on the 10% held-out test set. This approach ensured fair model comparison while avoiding data leakage. Supplementary Table 1 summarizes the hyperparameter grids used for each classifier. For example, the random forest model was tuned over n_estimators (100, 200, 300) and max_depth (None, 10, 20), while XGBoost included learning_rate and max_depth parameters.

**1.3. Final Hyperparameters for the Remaining Classifiers**

A full list of the optimal hyperparameter settings for the remaining classifiers is provided below. The SVM was optimized with C = 1 and an ‘rbf’ kernel. The Logistic Regression model used C = 1 as the final regularization strength. The Naïve Bayes classifier did not require any hyperparameter tuning and was used with default settings.

**Supplementary Materials**

**Supplementary tables and legends.**

**Table S1. Inclusion and exclusion criteria for ECON, ROCK-D, and SPARK cohorts.**

| **Study** | **Inclusion Criteria** | **Exclusion Criteria** |
| --- | --- | --- |
| **ECON** | • Adults aged ≥18 years  • CKD stage 5D patients on maintenance hemodialysis for ≥3 months | • Diagnosis of malignancy requiring chemotherapy or with metastatic disease  • Absolute contraindications to CPET (recent major cardiac event, unstable angina, syncope, uncontrolled arrhythmias, acute heart failure, respiratory failure, or physical disability precluding safe and adequate testing)  • Currently pregnant or breastfeeding  • Active implanted medical devices (e.g., pacemaker, defibrillator)  • Weight > 300 pounds (≈136 kg) |
| **ROCK-D** | • Age ≥18 years  • All races/ethnicities, both genders  • CKD stage 5D patients on thrice-weekly hemodialysis for ≥3 months  • English or Spanish speaking patients | • Inability to exercise due to musculoskeletal issues  • Unlikely to complete all protocol-required visits/procedures  • Active implanted medical devices (e.g., pacemaker, defibrillator)  • Pregnancy or breastfeeding  • Contraindications to CPET per SOP  • Weight > 300 pounds (≈136 kg)  • Malignancy requiring chemotherapy or with metastatic disease |
| **SPARK (CKD group only)** | • CKD stage 5D patients on hemodialysis  • Receiving maintenance hemodialysis 3× weekly for ≥3 months  • Age ≥18 years  • All races, ethnicities, and genders  • English speaking patients  • Agreement to participate in FIT Core study (IRB #1707550885) | • Receiving home hemodialysis or peritoneal dialysis  • Malignancy requiring chemotherapy or with metastatic disease  • Unlikely to complete all study visits/procedures  • Pregnancy or breastfeeding  • Active implanted medical devices (e.g., pacemaker, defibrillator)  • Weight > 300 pounds (≈136 kg)  • Contraindications to CPET per SOP |

**Note.** The standard operating procedure (SOP) for cardiopulmonary exercise testing (CPET), including contraindications and safety measures, is detailed in the methodology paper by Lim et al. (CJASN 2025) (6).

**Table S2. Hyperparameter grid used for classifier optimization**

| **Classifiers** | **Hyperparameters** |
| --- | --- |
| Support Vector Machine (SVM) | C: [0.1, 1, 10] kernel: ['linear', 'rbf'] |
| Random Forest | n_estimators: [100, 200, 300] max_depth: [None, 10, 20] |
| Decision Tree | max_depth: [None, 10, 20] |
| K-Nearest Neighbors (KNN) | n_neighbors: [3, 5, 7] |
| Naïve Bayes | No tunable hyperparameters |
| Logistic Regression | C: [0.1, 1, 10] |
| XGBoost | n_estimators: [100, 200, 300] max_depth: [3, 5, 7] learning_rate: [0.01, 0.1, 0.2] |

**Note.** Values in brackets indicate the candidate values tested for each hyperparameter during the optimization process. For instance, for XGBoost, n_estimator: [100, 200, 300] means that models were trained using 100, 200, and 300 trees, respectively, and learning_rate: [0.01, 0.1, 0.2,] refers to the range of step size considered during boosting.

**Table S3. ML classifier performance on GLCM-processed distal tibia cortical compartment on the held-out test set.**

| **GLCM** | ***GLCM & Distal Tibia Cortical Bone Classification Performance Matrics*** | | | | | | |  |
| --- | --- | --- | --- | --- | --- | --- | --- | --- |
|  | **Sensitivity** | **Specificity** | **FNR** | **PPV** | **NPV** | **Accuracy** | **AUC-ROC** | |
| **SVM** | 0.748 | 0.630 | 0.252 | 0.651 | 0.730 | 0.686 | 0.772 | |
| **RF** | 0.868 | 0.921 | 0.132 | 0.911 | 0.883 | 0.896 | 0.971 | |
| **DT** | 0.852 | 0.874 | 0.148 | 0.861 | 0.865 | 0.863 | 0.868 | |
| **KNN** | 0.672 | 0.689 | 0.328 | 0.666 | 0.695 | 0.681 | 0.751 | |
| **NB** | 0.558 | 0.888 | 0.442 | 0.821 | 0.685 | 0.730 | 0.795 | |
| **LR** | 0.680 | 0.792 | 0.320 | 0.751 | 0.728 | 0.738 | 0.834 | |
| **XGBoost** | 0.875 | 0.924 | 0.125 | 0.914 | 0.889 | 0.900 | 0.974 | |

Note. The FNRs were directly computed from the confusion matrices using the formula FN / (FN + TP), not by subtracting the rounded sensitivity values, such as RF, DT, and XGBoost, ensuring greater numerical accuracy than using 1 – sensitivity derived from the table. SVM = Support Vector Machine; RF = Random Forest; DT = Decision Tree; KNN = K-nearest neighbors; NB = Navie Bayes; LR = Logistic Regression; XGBoost = eXtreme Gradient Boosting; AUC-ROC = area under the curve of the receiver operating characteristic; FNR = False Negative Rate; PPV = Positive Predictive Value; NPV = Negative Predictive Value.

**Table S4. ML classifier performance on LBP-processed distal tibia cortical compartment on the held-out test set.**

| **LBP** | ***LBP & Distal Tibia Cortical Bone Classification Performance Matrics*** | | | | | | |  |
| --- | --- | --- | --- | --- | --- | --- | --- | --- |
|  | **Sensitivity** | **Specificity** | **FNR** | **PPV** | **NPV** | **Accuracy** | **AUC-ROC** | |
| **SVM** | 0.564 | 0.591 | 0.436 | 0.560 | 0.595 | 0.578 | 0.632 | |
| **RF** | 0.987 | 0.986 | 0.013 | 0.985 | 0.988 | 0.986 | 0.999 | |
| **DT** | 0.942 | 0.943 | 0.058 | 0.938 | 0.947 | 0.943 | 0.944 | |
| **KNN** | 0.980 | 0.978 | 0.019 | 0.976 | 0.982 | 0.979 | 0.999 | |
| **NB** | 0.809 | 0.376 | 0.191 | 0.545 | 0.681 | 0.584 | 0.766 | |
| **LR** | 0.413 | 0.724 | 0.587 | 0.579 | 0.572 | 0.574 | 0.629 | |
| **XGBoost** | 0.988 | 0.990 | 0.012 | 0.989 | 0.989 | 0.989 | 0.999 | |

Note. The FNRs were directly computed from the confusion matrices using the formula FN / (FN + TP), not by subtracting the rounded sensitivity values, such as RF, KNN, and XGBoost, ensuring greater numerical accuracy than using 1 – sensitivity derived from the table. SVM = Support Vector Machine; RF = Random Forest; DT = Decision Tree; KNN = K-nearest neighbors; NB = Navie Bayes; LR = Logistic Regression; XGBoost = eXtreme Gradient Boosting; AUC-ROC = area under the curve of the receiver operating characteristic; FNR = False Negative Rate; PPV = Positive Predictive Value; NPV = Negative Predictive Value.

**Table S5. ML classifier performance on GLCM-processed diaphyseal tibia cortical compartment on the held-out test set.**

| **GLCM** | ***GLCM & Diaphyseal Tibia Cortical Bone Classification Performance Matrics*** | | | | | | |  |
| --- | --- | --- | --- | --- | --- | --- | --- | --- |
|  | **Sensitivity** | **Specificity** | **FNR** | **PPV** | **NPV** | **Accuracy** | **AUC-ROC** | |
| **SVM** | 0.773 | 0.685 | 0.227 | 0.693 | 0.766 | 0.727 | 0.799 | |
| **RF** | 0.967 | 0.979 | 0.033 | 0.977 | 0.970 | 0.973 | 0.996 | |
| **DT** | 0.949 | 0.948 | 0.050 | 0.943 | 0.953 | 0.948 | 0.948 | |
| **KNN** | 0.734 | 0.777 | 0.266 | 0.752 | 0.760 | 0.756 | 0.832 | |
| **NB** | 0.711 | 0.608 | 0.289 | 0.626 | 0.696 | 0.658 | 0.761 | |
| **LR** | 0.684 | 0.781 | 0.316 | 0.742 | 0.728 | 0.734 | 0.783 | |
| **XGBoost** | 0.972 | 0.986 | 0.028 | 0.984 | 0.975 | 0.979 | 0.997 | |

Note. The FNRs were directly computed from the confusion matrices using the formula FN / (FN + TP), not by subtracting the rounded sensitivity values, such as RF, DT, and XGBoost, ensuring greater numerical accuracy than using 1 – sensitivity derived from the table. SVM = Support Vector Machine; RF = Random Forest; DT = Decision Tree; KNN = K-nearest neighbors; NB = Navie Bayes; LR = Logistic Regression; XGBoost = eXtreme Gradient Boosting; AUC-ROC = area under the curve of the receiver operating characteristic; FNR = False Negative Rate; PPV = Positive Predictive Value; NPV = Negative Predictive Value.

**Table S6. ML classifier performance on LBP-processed tibia diaphyseal cortical compartment on the held-out test set.**

| **LBP** | ***GLCM & Tibia Diaphyseal Cortical Bone Classification Performance Matrics*** | | | | | | |  |
| --- | --- | --- | --- | --- | --- | --- | --- | --- |
|  | **Sensitivity** | **Specificity** | **FNR** | **PPV** | **NPV** | **Accuracy** | **AUC-ROC** | |
| **SVM** | 0.704 | 0.475 | 0.296 | 0.553 | 0.635 | 0.585 | 0.651 | |
| **RF** | 0.991 | 0.994 | 0.009 | 0.994 | 0.991 | 0.993 | 0.999 | |
| **DT** | 0.956 | 0.976 | 0.044 | 0.974 | 0.960 | 0.967 | 0.966 | |
| **KNN** | 0.995 | 0.985 | 0.005 | 0.984 | 0.995 | 0.990 | 0.998 | |
| **NB** | 0.820 | 0.349 | 0.180 | 0.538 | 0.678 | 0.575 | 0.627 | |
| **LR** | 0.434 | 0.692 | 0.566 | 0.565 | 0.570 | 0.568 | 0.629 | |
| **XGB** | 0.995 | 0.993 | 0.005 | 0.992 | 0.995 | 0.994 | 0.999 | |

Note. The FNRs were directly computed from the confusion matrices using the formula FN / (FN + TP), not by subtracting the rounded sensitivity values, such as RF, KNN, and XGBoost, ensuring greater numerical accuracy than using 1 – sensitivity derived from the table. SVM = Support Vector Machine; RF = Random Forest; DT = Decision Tree; KNN = K-nearest neighbors; NB = Navie Bayes; LR = Logistic Regression; XGBoost = eXtreme Gradient Boosting; AUC-ROC = area under the curve of the receiver operating characteristic; FNR = False Negative Rate; PPV = Positive Predictive Value; NPV = Negative Predictive Value.

**Table S7. Patient-level classification performance across feature extractors and machine-learning classifiers at distal and diaphyseal tibial sites.**

| **Classifier** | **Distal Tibia** | | | **Diaphyseal Tibia** | | |
| --- | --- | --- | --- | --- | --- | --- |
|  | **AUC (95% CI)** | **Sensitivity** | **Specificity** | **AUC (95% CI)** | **Sensitivity** | **Specificity** |
| **GLCM** | | | | | | |
| SVM | 0.875 (0.500–1.000) | 0.500 | 0.750 | 0.750 (0.286–1.000) | 0.750 | 0.500 |
| Random Forest | 1.000 (1.000–1.000) | 1.000 | 0.750 | 0.438 (0.000–0.885) | 0.750 | 0.250 |
| Decision Tree | 0.875 (0.625–1.000) | 1.000 | 0.750 | 0.625 (0.250–0.929) | 0.750 | 0.500 |
| KNN | 1.000 (1.000–1.000) | 1.000 | 1.000 | 0.688 (0.233–1.000) | 0.750 | 0.500 |
| Naïve Bayes | 1.000 (1.000–1.000) | 1.000 | 0.500 | 0.812 (0.383–1.000) | 0.750 | 0.500 |
| Logistic Regression | 0.875 (0.500–1.000) | 0.750 | 0.750 | 0.750 (0.286–1.000) | 0.750 | 0.750 |
| XGBoost | 0.938 (0.667–1.000) | 1.000 | 0.750 | 0.562 (0.000–1.000) | 0.500 | 0.500 |
| **LBP** | | | | | | |
| SVM | 0.125 (0.000–0.500) | 0.000 | 1.000 | 0.188 (0.000–0.667) | 0.000 | 1.000 |
| Random Forest | 1.000 (1.000–1.000) | 1.000 | 0.500 | 0.688 (0.200–1.000) | 0.750 | 0.750 |
| Decision Tree | 0.875 (0.625–1.000) | 0.750 | 1.000 | 0.875 (0.625–1.000) | 1.000 | 0.750 |
| KNN | 0.469 (0.067–0.875) | 0.750 | 0.250 | 0.531 (0.000–1.000) | 0.750 | 0.500 |
| Naïve Bayes | 0.812 (0.333–1.000) | 0.750 | 0.250 | 0.750 (0.250–1.000) | 1.000 | 0.500 |
| Logistic Regression | 0.875 (0.500–1.000) | 0.000 | 1.000 | 0.812 (0.333–1.000) | 0.000 | 1.000 |
| XGBoost | 1.000 (1.000–1.000) | 1.000 | 1.000 | 0.875 (0.467–1.000) | 1.000 | 0.000 |
| **GLCM + LBP** | | | | | | |
| SVM | 0.875 (0.500–1.000) | 0.500 | 0.750 | 0.750 (0.286–1.000) | 0.750 | 0.500 |
| Random Forest | 0.938 (0.729–1.000) | 1.000 | 0.750 | 0.625 (0.125–1.000) | 0.750 | 0.750 |
| Decision Tree | 0.625 (0.500–0.875) | 1.000 | 0.250 | 0.625 (0.250–0.929) | 0.750 | 0.500 |
| KNN | 1.000 (1.000–1.000) | 1.000 | 1.000 | 0.688 (0.233–1.000) | 0.750 | 0.500 |
| Naïve Bayes | 1.000 (1.000–1.000) | 1.000 | 0.250 | 0.812 (0.400–1.000) | 1.000 | 0.250 |
| Logistic Regression | 0.875 (0.500–1.000) | 0.750 | 0.750 | 0.750 (0.286–1.000) | 0.750 | 0.750 |
| XGBoost | 1.000 (1.000–1.000) | 1.000 | 0.750 | 0.625 (0.125–1.000) | 0.750 | 0.250 |

Note. Patient-level performance was derived by aggregating radiomic features per patient (mean across all slices) prior to classification. Data were partitioned at the patient level using an 80/10/10 split (training/validation/test), yielding approximately 57 training, 7 validation, and 7 test patients. Confidence intervals (CI) are wide due to the small test set size (n ≈ 7–8), which reflects the limited statistical precision associated with the small patient-level test set. Perfect AUC values (1.000) with narrow bootstrap confidence intervals reflect the small test cohort (n ≈ 7–8 patients) and should not be interpreted as evidence of definitive classifier generalizability. AUC = area under the receiver operating characteristic curve; CI = 95% bootstrap confidence interval (2,000 resamples); SVM = Support Vector Machine; KNN = K-Nearest Neighbors; NB = Naïve Bayes; LR = Logistic Regression; RF = Random Forest; GLCM = Gray-Level Co-occurrence Matrix; LBP = Local Binary Pattern.

**Supplementary figures and legends.**

Note. The held-out test set evaluates how well the trained model performs on entirely new, unseen data, providing an estimate of its real-world predictive ability.

**Figure S1. Confusion Matrix for binary (non-CKD, CKD) classification using the top three ML classifiers from (A) Random Forest and (B) Decision Tree to (C) XGBoost based on distal tibia cortical bone data using GLCM features on the held-out test set.** The number within each box represents the number of images classified.

Note. The held-out test set evaluates how well the trained model performs on entirely new, unseen data, providing an estimate of its real-world predictive ability.

**Figure S2. Confusion Matrix for binary (non-CKD, CKD) classification using the top three ML classifiers from (A) Random Forest and (B) KNN to (C) XGBoost based on distal tibia cortical bone data using LBP on the held-out test set.** The number within each box represents the number of images classified.

Note. The held-out test set evaluates how well the trained model performs on entirely new, unseen data, providing an estimate of its real-world predictive ability.

**Figure S3. Confusion Matrix for binary (non-CKD, CKD) classification using the top three ML classifiers from (A) Random Forest and (B) Decision Tree to (C) XGBoost based on diaphyseal tibia cortical bone data using GLCM features on the held-out test set.** The number within each box represents the number of images classified.

Note. The held-out test set evaluates how well the trained model performs on entirely new, unseen data, providing an estimate of its real-world predictive ability.

**Figure S4. Confusion Matrix for binary (non-CKD, CKD) classification using the top three ML classifiers from (A) Random Forest and (B) KNN to (C) XGBoost based on diaphyseal tibia cortical bone data using LBP on the held-out test set.** The number within each box represents the number of images classified.

Note. SVM = Support Vector Machine; RF = Random Forest; DT = Decision Tree; KNN = K-nearest neighbors; NB = Navie Bayes; LR = Logistic Regression; XGBoost = eXtreme Gradient Boosting; AUC-ROC = area under the curve of the receiver operating characteristic. GLCM: Gray Level Co-Occurrence.

**Figure S5. Area Under the receiver operating characteristic (AUC-ROC) curves for discriminating CKD patients from non-CKD participants HR-pQCT distal tibia cortical images on the held-out test set.** The AUC-ROC curves were derived from diayphseal tibia cortical bone data inputted various classification models ranging from SVM to XGBoost combined the specific radimics technique, **GLCM features**, based on transfer deep learning with model acceleration.

Note. SVM = Support Vector Machine; RF = Random Forest; DT = Decision Tree; KNN = K-nearest neighbors; NB = Navie Bayes; LR = Logistic Regression; XGBoost = eXtreme Gradient Boosting; AUC-ROC = area under the curve of the receiver operating characteristic. LBP = Local Binary Pattern.

**Figure S6. Area Under the receiver operating characteristic (AUC-ROC) curves for discriminating CKD patients from non-CKD participants in HR-pQCT distal tibia cortical images on the held-out test set.** The AUC-ROC curves were derived from distal tibia cortical bone data inputted various classification models ranging from SVM to XGBoost combined the specific radimics technique, **LBP features**, based on transfer deep learning with model acceleration.

Note. SVM = Support Vector Machine; RF = Random Forest; DT = Decision Tree; KNN = K-nearest neighbors; NB = Navie Bayes; LR = Logistic Regression; XGBoost = eXtreme Gradient Boosting; AUC-ROC = area under the curve of the receiver operating characteristic. GLCM: Gray Level Co-Occurrence.

**Figure S7. Area Under the receiver operating characteristic (AUC-ROC) curves for discriminating CKD patients from non-CKD participants in HR-pQCT diaphyseal tibia cortical images on the held-out test set.** The AUC-ROC curves were derived from diayphseal tibia cortical bone data inputted various classification models ranging from SVM to XGBoost combined the specific radimics technique, **GLCM features**, based on transfer deep learning with model acceleration.

Note. SVM = Support Vector Machine; RF = Random Forest; DT = Decision Tree; KNN = K-nearest neighbors; NB = Navie Bayes; LR = Logistic Regression; XGBoost = eXtreme Gradient Boosting; AUC-ROC = area under the curve of the receiver operating characteristic. LBP = Local Binary Pattern.

**Figure S8. Area Under the receiver operating characteristic (AUC-ROC) curves for discriminating CKD patients from non-CKD participants in HR-pQCT diaphyseal tibia cortical images on the held-out test set.** The AUC-ROC curves were derived from diaphyseal tibia cortical bone data inputted various classification models ranging from SVM to XGBoost combined the specific radimics technique, **LBP features**, based on transfer deep learning with model acceleration.

**Figure S9. Visual inspection of misclassified samples between non-CKD and CKD HR-pQCT distal tibia images, resulting from similar characteristics as identified by the overall superior model (XGBoost) combined GLCM with LBP.** Each subgroup (non-CKD and CKD) is represented by a misclassified axial cross-section from the distal tibia site. In total, nine CKD images were misclassified as non-CKD. The model correctly classified all 1,259 non-CKD images, indicating that no non-CKD images were misclassified, while 9 of 1,161 CKD images were misclassified as non-CKD.

**Figure S10. Visual inspection of misclassified samples between non-CKD and CKD HR-pQCT diaphyseal tibia images, resulting from similar characteristics as identified by the overall superior model (XGBoost) combined GLCM with LBP.** Representative axial cross-sections illustrate misclassified cases at the diaphyseal tibia site. In total, one CKD image was misclassified as non-CKD (A), and two non-CKD images were misclassified as CKD (B). Overall, the model correctly classified 1,257 of 1,259 non-CKD images.

**Figure S11. Patient-level ROC curves for discriminating CKD patients from non-CKD participants using HR-pQCT cortical bone radiomics.** Results are shown for (A) distal tibia – GLCM, (B) distal tibia – LBP, (C) distal tibia – GLCM+LBP, (D) diaphyseal tibia – GLCM, (E) diaphyseal tibia – LBP, and (F) diaphyseal tibia – GLCM+LBP. AUC = area under the receiver operating characteristic curve; GLCM = gray-level co-occurrence matrix; LBP = local binary pattern; SVM = Support Vector Machine; RF = Random Forest; DT = Decision Tree; KNN = K-nearest neighbors; NB = Naïve Bayes; LR = Logistic Regression.

**REFRENCES**

1.	Warden SJ, Liu Z, Fuchs RK, van Rietbergen B, Moe SM. Reference data and calculators for second-generation HR-pQCT measures of the radius and tibia at anatomically standardized regions in White adults. Osteoporosis International. 2022;33(4):791-806.

2.	Bonaretti S, Majumdar S, Lang TF, Khosla S, Burghardt AJ. The comparability of HR-pQCT bone measurements is improved by scanning anatomically standardized regions. Osteoporosis International. 2017;28:2115-28.

3.	Arias-Moreno AJ, Hosseini HS, Bevers M, Ito K, Zysset P, van Rietbergen B. Validation of distal radius failure load predictions by homogenized-and micro-finite element analyses based on second-generation high-resolution peripheral quantitative CT images. Osteoporosis international. 2019;30:1433-43.

4.	Stevens LA, Coresh J, Greene T, Levey AS. Assessing kidney function—measured and estimated glomerular filtration rate. New England Journal of Medicine. 2006;354(23):2473-83.

5.	Lerman P. Fitting segmented regression models by grid search. Journal of the Royal Statistical Society Series C: Applied Statistics. 1980;29(1):77-84.

6.	Arroyo E, Groninger NW, Dillman DE, Burney HN, Li X, Li Y, et al. Abnormal Cardiovascular Functional Responses After the Long Interdialytic Interval: A Quasi-Experimental Crossover Study. Clinical Journal of the American Society of Nephrology. 2025:10.2215.
